# Triple-positive non-small cell lung cancer harboring EGFR mutation, ALK rearrangement, and high PD-L1 expression: a case report and literature review

**DOI:** 10.3389/fonc.2025.1727899

**Published:** 2026-01-27

**Authors:** Mengjie Mao, Qingxiu Tao, Zhuo Zuo, Jun Ge, Yuke Tian, Bin Liu

**Affiliations:** 1 School of Medical and Life Sciences, Chengdu University of Traditional Chinese Medicine, Chengdu, China; 2 School of Medicine, University of Electronic Science and Technology of China, Chengdu, China; 3 Department of Pathology, Sichuan Cancer Hospital & Institute, Sichuan Cancer Center, Affiliated Cancer Hospital of University of Electronic Science and Technology of China, Chengdu, China; 4 Department of Medical Oncology, Sichuan Cancer Hospital and Institute, Sichuan Cancer Center, Affiliated Cancer Hospital of University of Electronic Science and Technology of China, Chengdu, China

**Keywords:** ALK rearrangement, co-mutation, EGFR mutation, non-small cell lung cancer, PD-L1, sequential targeted therapy

## Abstract

**Background:**

Epidermal growth factor receptor (EGFR) mutations and anaplastic lymphoma kinase (ALK) rearrangements are typically considered mutually exclusive in non-small cell lung cancer (NSCLC). However, rare cases with coexisting EGFR/ALK alterations and high programmed death ligand-1 (PD-L1) expression, termed “triple-positive” NSCLC, have been reported. Optimal treatment strategies for this unique subgroup remain undefined.

**Case presentation:**

We describe a 54-year-old woman with stage IV lung adenocarcinoma harboring an EGFR exon 19 deletion, ALK–EML4/KIF5B fusion (V3a/b), and high PD-L1 expression (TPS = 90%). The patient received first-line osimertinib combined with pemetrexed/cisplatin, achieving durable disease control for 17 months. Upon progression, rebiopsy and next-generation sequencing (NGS) revealed persistent ALK fusion and newly acquired ERBB2 amplification. Treatment was switched to alectinib, leading to significant tumor regression and partial response.

**Conclusion:**

This case illustrates that in triple-positive NSCLC, initial EGFR-TKI combined with chemotherapy can achieve long-term control, while dynamic molecular profiling at progression is essential for identifying resistance mechanisms. Sequential targeted therapy guided by NGS remains a cornerstone for precision management in this complex molecular subtype.

## 1 Introduction

Lung cancer is the leading cause of cancer-related morbidity and mortality worldwide. With the advent of precision oncology, EGFR mutations and ALK rearrangements have become two of the most important molecular drivers in NSCLC. Traditionally, these mutations were regarded as mutually exclusive; however, increasing evidence shows that they can coexist within the same tumor, creating a rare and clinically challenging molecular subtype.

For this rare combination, our case adds novel insights regarding the role of high PD-L1 expression (TPS = 90%) and how resistance mechanisms evolve over time, particularly with the emergence of ERBB2 amplification. This highlights the importance of dynamic molecular profiling, especially during disease progression, to guide subsequent therapy choices. The decision is further complicated when PD-L1 expression is considered, as it strongly influences immunotherapy use. Nevertheless, in the presence of classical driver mutations, ICIs generally provide limited benefit, even with high PD-L1 expression.

Herein, we report a rare case of triple-positive advanced lung adenocarcinoma with EGFR exon 19 deletion, ALK-EML4/KIF5B fusion, and high PD-L1 expression. We describe the patient’s clinical course, the therapeutic decisions made, and the role of dynamic molecular profiling in guiding sequential targeted therapy.

## 2 Case presentation

**Figure 1:**
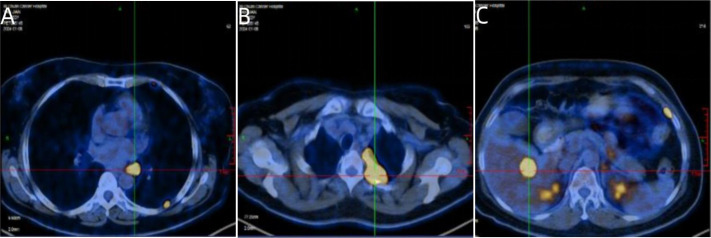
Representative PET-CT images at initial diagnosis. **(A)** Axial PET-CT image showing enlarged left hilar lymph nodes. **(B)** Axial PET-CT image demonstrating multiple pleural nodules along the left pleura. **(C)** Axial PET-CT image revealing a hepatic metastatic lesion in the right lobe of the liver.

**Figure 2:**
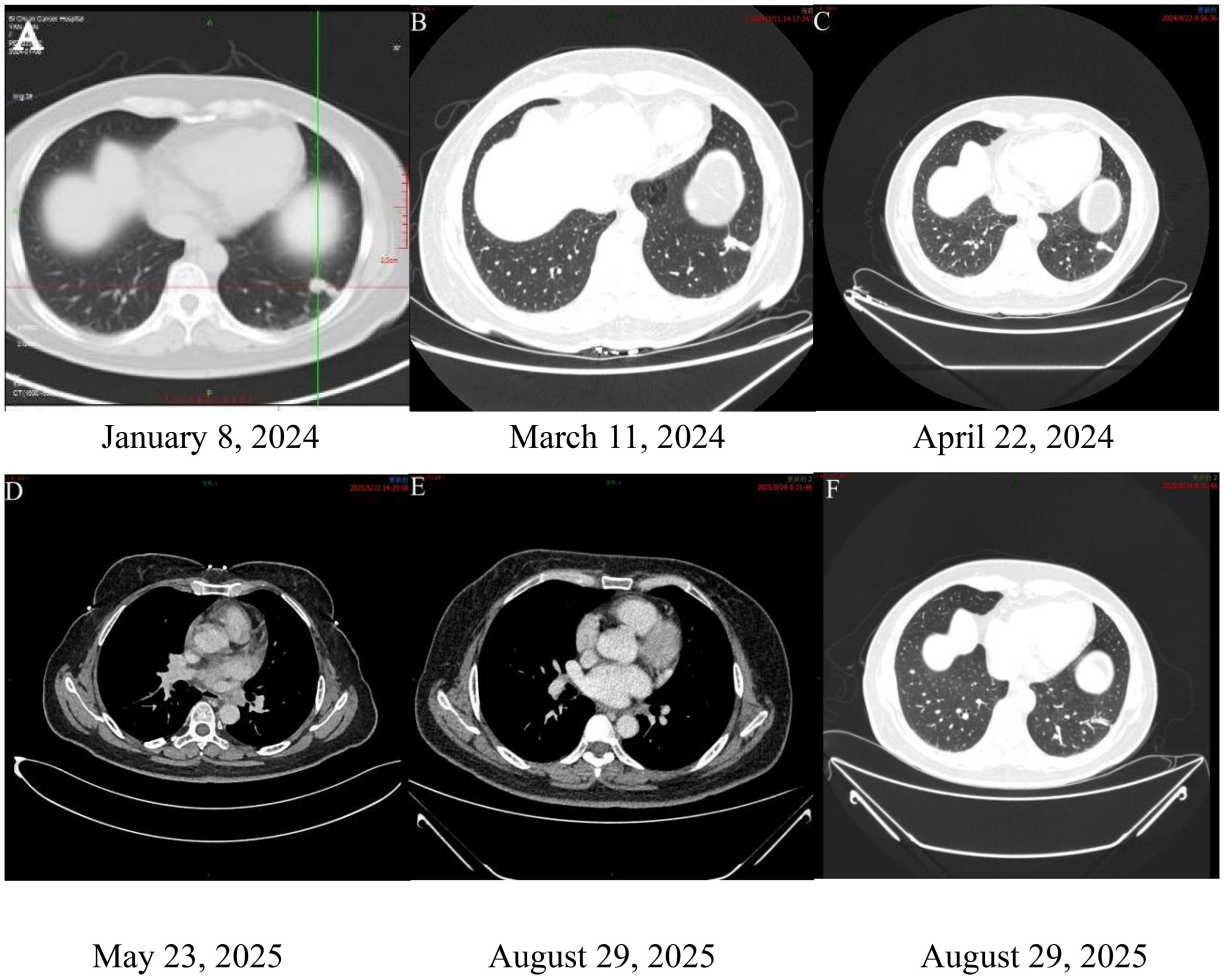
Radiological findings of the patient during the clinical course. **(A)** Baseline PET-CT showing a solid nodule in the outer basal segment of the left lower lobe, enlarged left hilar lymph nodes, and multiple pleural nodules and masses. **(B, C)** CT scans after treatment with osimertinib plus chemotherapy showing stable disease. **(D)** At disease progression, CT demonstrated a solid nodule in the left lower lobe and new soft tissue thickening in the left hilum. **(E, F)** Follow-up CT after alectinib treatment showing significant reduction in the left lower lobe nodule and hilar soft tissue thickening. Therapeutic timeline of the patient is shown in **Figure 5**.

A 54-year-old woman was admitted in December 2023 after a routine health examination revealed a mass in the left lower lobe of the lung. Chest computed tomography (CT) showed a round, soft tissue nodule in the outer basal segment of the left lower lobe, accompanied by multiple nodules and masses in the left pleura. Specifically, the primary nodule in the outer basal segment measured approximately 2.2 cm in diameter, while the largest pleural nodule along the fissure demonstrated a long-axis diameter of about 3.8 cm. She was referred to Sichuan Cancer Hospital for further evaluation. Positron emission tomography.dy (PET-CT) confirmed a solid nodule in the left lower lobe with high suspicion of primary lung cancer. Enlarged left hilar lymph nodes, multiple pleural nodules, and a hepatic lesion were also detected, consistent with metastatic disease as shown in **Figure 1**. Additional findings included possible left adrenal hyperplasia and a soft tissue lesion in the left parotid gland, which was recommended for follow-up. No other abnormalities were observed (**Figure 2A**).

**Figure 3:**
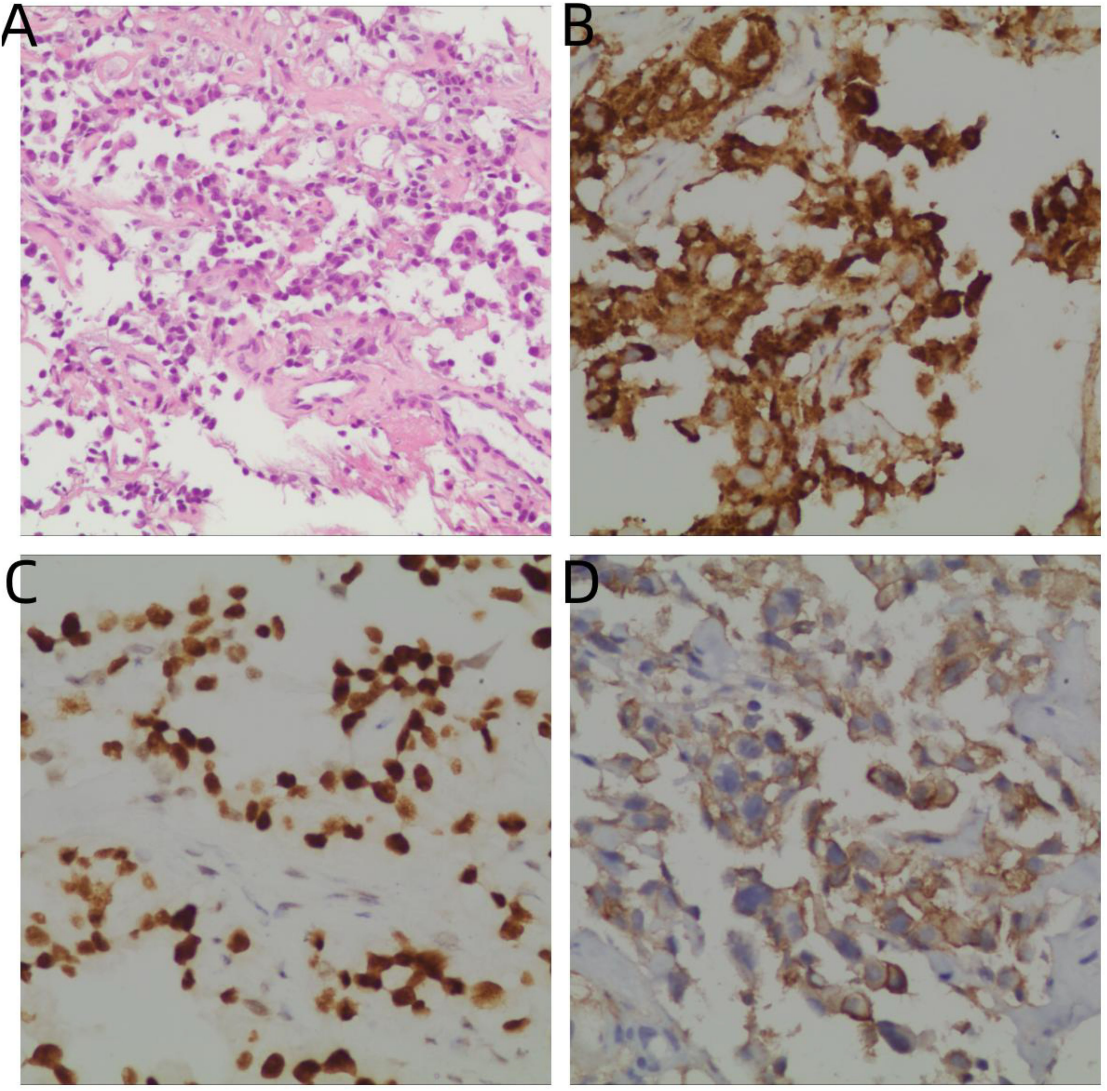
Histopathological findings at initial diagnosis. **(A)** Hematoxylin–eosin staining showing glandular adenocarcinoma morphology (×200). **(B)** Cytoplasmic granular positivity for Napsin A (IHC, ×400). **(C)** Tumor cells showing diffuse nuclear positivity for TTF-1 (IHC, ×400). **(D)** Strong membranous PD-L1 expression with a tumor proportion score (TPS) of 90% (IHC, ×400).

An ultrasound-guided biopsy of the pulmonary lesion confirmed adenocarcinoma (**Figure 3**). Immunohistochemistry showed CK7(+), TTF-1(+), NapsinA(+), Ki67 (~10%), CK5/6(-), P40(-), and high PD-L1 expression (TPS = 90%). Next-generation sequencing (NGS) identified an EGFR exon 19 deletion and an ALK-EML4/KIF5B fusion. The final diagnosis was stage IV (cT4N2M1) lung adenocarcinoma with hilar, pleural, and hepatic metastases. On January 17, 2024, first-line treatment was initiated with osimertinib combined with pemetrexed/cisplatin chemotherapy.

**Figure 4:**
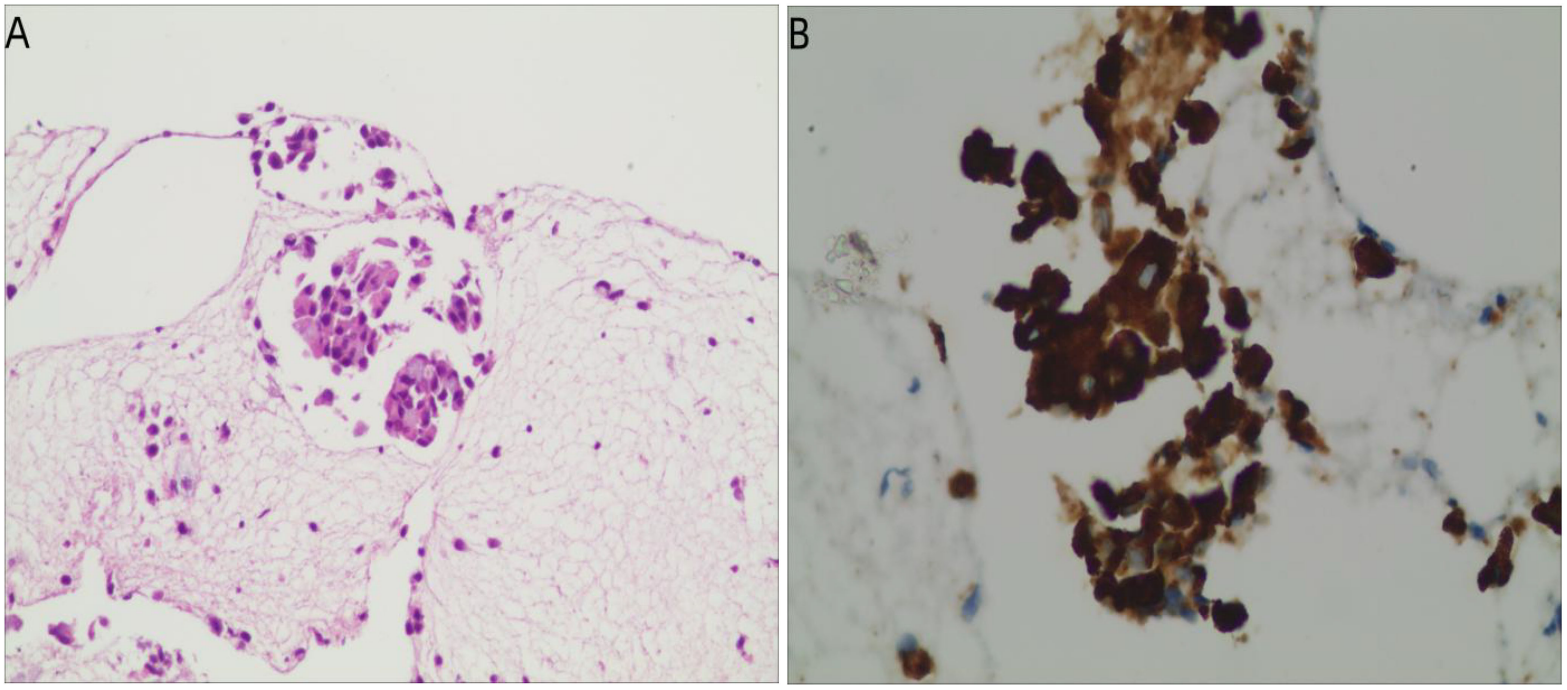
Histopathological findings at after diagnosis. **(A)** Hematoxylin–eosin staining of the rebiopsy specimen at disease progression showing recurrent adenocarcinoma morphology (×200). **(B)** Immunohistochemical staining for ALK (D5F3) showing strong cytoplasmic positivity in tumor cells of the recurrent lesion (×400).

Follow-up and response: After 15 weeks of therapy (April 22, 2024), chest CT revealed stable disease (SD) (**Figures 2B**, **1C**). The patient subsequently received 14 cycles of maintenance pemetrexed (10 cycles combined with cisplatin followed by 4 cycles of pemetrexed disodium) while continuing osimertinib. She maintained disease stability for 76 weeks (approximately 17 months).On July 3, 2025, CT demonstrated new soft tissue thickening in the left hilar region, consistent with progressive disease (PD) (**Figure 2D**). Endobronchial ultrasound-guided biopsy confirmed recurrent adenocarcinoma. Immunohistochemistry showed CK7(+), TTF-1(+), NapsinA(+), Ki67 (~5%), CK5/6 (focal+), P40(–), and BRG1/SMARCA4(+) (**Figure 4**). 

**Figure 5:**
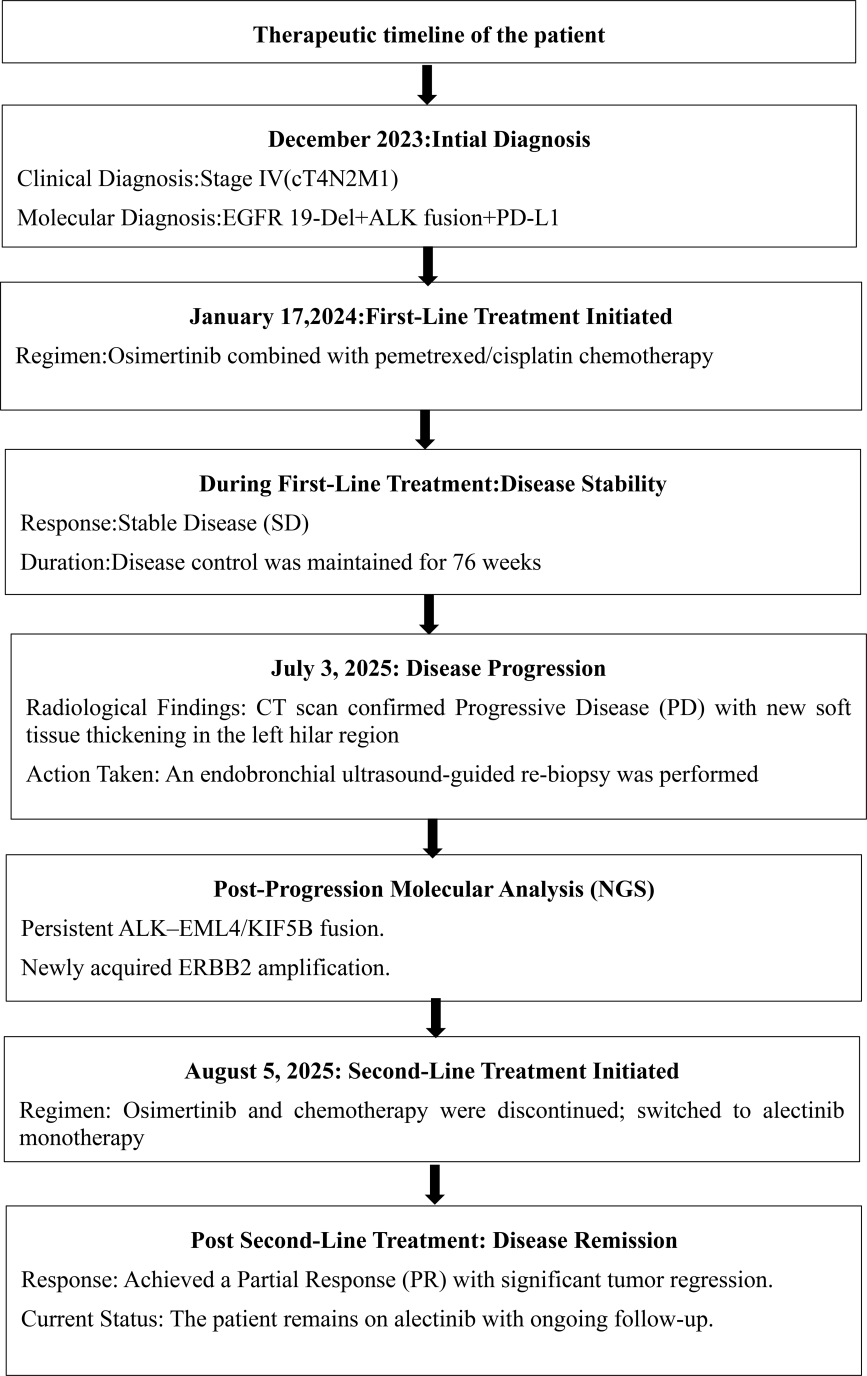
Therapeutic timeline of the patient.

Molecular analysis at progression: To investigate resistance mechanisms, the tumor specimen was submitted to Genecast (CAP/CLIA-certified laboratory) for targeted NGS using the Illumina NovaSeq 6000 platform (average depth ~1000×, >95% coverage ≥200×). Testing revealed persistent ALK–EML4/KIF5B fusion (V3a/b; E6:A20, allele frequency 6.42%) and ERBB2 amplification, suggesting a shift in resistance mechanisms. The patient’s genetic testing results as shown in **Table 1**. Second-line treatment: On August 5, 2025, chemotherapy and osimertinib were discontinued, and the patient was started on alectinib. A follow-up CT on August 29, 2025 showed marked tumor regression: the left lower lobe nodule decreased from 2.5 × 2.0 cm to 0.8 cm, and the left hilar soft tissue thickening was significantly reduced (**Figures 2E, F**). The overall treatment course and sequencing of therapies are summarized in the therapeutic timeline (**Figure 5**). No intracranial metastases were observed. The patient achieved a partial response (PR) and remains on alectinib with ongoing follow-up.

**Table 1:** Genetic testing results of the patient.

Gene name	Nucleotide variation	Result	After Osimertinib Treatment
EGFR	19-Del T790M	Positive Negative	Negative Negative
ALK	EML4/KIF5B	Positive	Positive
ERBB2	–	Negative	Positive
ROS1	–	Negative	Negative
RET	–	Negative	Negative
RAS	K-Ras Exon-2 N-Ras Exon-3	Negative	Negative
Braf	V600E	Negative	Negative
PIK3CA	Exon-9,Exon-20	Negative	Negative
HER2	Exon-20	Negative	Negative
MET	Exon-14	Negative	Negative

## 3 Discussion

Lung cancer remains the leading cause of cancer-related incidence and mortality worldwide (1). NSCLC is the predominant histological subtype, comprising approximately 85% of cases (2). In the present case, the patient was clinically staged as cT4N2M1 based on imaging findings, including enlarged left hilar lymph nodes and possible mediastinal involvement on PET-CT. Although pathological confirmation of mediastinal nodal metastasis was not obtained, the clinical and radiological evidence supported an N2 designation according to the 8th edition TNM staging system. This highlights the importance of integrating imaging and clinical assessment in staging, particularly when invasive nodal sampling is not feasible. Activating mutations in the EGFR gene and rearrangements of the ALK gene are key oncogenic drivers in NSCLC. While traditionally considered mutually exclusive (3), emerging evidence confirms that concurrent EGFR mutations and ALK alterations can occur (4), with a reported incidence of 0.06% to 1.6%, varying by detection methodology and patient population (5). EGFR tyrosine kinase inhibitors (EGFR-TKIs) effectively block downstream signaling by inhibiting EGFR activity, demonstrating superior efficacy over chemotherapy in patients with solitary EGFR mutations (6, 7). Following the landmark FLAURA trial (8), the third-generation TKI osimertinib, which improves overall survival, has become the standard of care for the first-line treatment of EGFR-mutant NSCLC. In parallel, ALK rearrangements result in the aberrant activation of the ALK kinase domain, which promotes tumor cell proliferation via downstream pathways (9). The most common fusion variant, EML4-ALK, produces a fusion protein that drives constitutive ALK phosphorylation and malignant transformation (10). Alectinib, a highly selective second-generation ALK-TKI with excellent central nervous system permeability, is the established first-line therapy for patients with ALK-positive advanced NSCLC.

For the rare cohort of NSCLC patients harboring concurrent EGFR and ALK mutations, the optimal treatment strategy is undefined, with no clinical guidelines to direct the sequencing of EGFR and ALK-TKIs. The primary therapeutic approaches are as follows:

1. EGFR-TKI monotherapy: One prevailing strategy is to identify and target the “dominant” driver mutation. Given the higher prevalence of EGFR mutations and the longer developmental history of its inhibitors, the EGFR pathway is often presumed to be the primary oncogenic driver. However, one study reported (11) that EGFR-TKIs were ineffective in 10 patients with EML4-ALK fusions, who conversely responded favorably to pemetrexed. Furthermore, research has shown that (12) first-line ametenib plus pemetrexed and carboplatin yields high response rates in EGFR-mutant NSCLC. In line with this, the patient in this case was initially treated with osimertinib, pemetrexed, and cisplatin. This regimen was effective, achieving disease stability for 76 weeks (approx. 17.5 months).However, the efficacy of EGFR-TKIs in this co-mutated population is inconsistent. A large study (13) involving nearly 3,000 patients confirmed that those with concurrent EGFR and ALK alterations had a significantly shorter median progression-free survival when treated with EGFR-TKIs compared to patients with solitary EGFR mutations, underscoring the role of ALK rearrangement as an intrinsic resistance mechanism, suggesting that ALK rearrangement may function as an intrinsic resistance mechanism. The eventual disease progression in our patient underscores the limitation that inhibiting the EGFR pathway alone may be insufficient for durable tumor control.
2. ALK-TKI monotherapy: An alternative approach is to prioritize the ALK pathway, which a growing body of evidence suggests may be the more potent oncogenic driver in some co-mutated tumors. A retrospective analysis (14) of over 100 patients with dual mutations provided comparative data, indicating that patients receiving ALK-TKIs achieved numerically higher objective response and disease control rates than those treated with EGFR-TKIs. The clinical course of our patient perfectly illustrates this principle. Upon progression on first-line osimertinib and chemotherapy, repeat NGS confirmed the persistence of the ALK fusion as the likely driver of resistance, alongside a newly acquired ERBB2 amplification. Consequently, the treatment was decisively switched to the potent ALK inhibitor alectinib. This resulted in a rapid and significant shrinkage of lung lesions, achieving a partial response (PR). This dramatic clinical reversal provided compelling evidence that the ALK pathway had emerged as the dominant oncogenic driver following the failure of osimertinib.
3. Concurrent or sequential TKI therapy: A third strategy involves the concurrent or sequential administration of both EGFR and ALK-TKIs. Case reports have documented successful outcomes (15, 16); for example (17), a patient with stage IV NSCLC with dual mutations achieved a PR with a second-line ALK-TKI plus local radiotherapy after progressing on first-line osimertinib. A retrospective analysis (18) systematically evaluated treatment outcomes in this population and concluded that both EGFR-TKIs and ALK-TKIs (e.g., crizotinib) exhibit clinical efficacy. Notably, this study proposed that a sequential TKI strategy, leveraging the activity of both drug classes, represents a rational and effective management approach for these complex cases. Our case, featuring a successful transition from first-line osimertinib-based therapy to second-line alectinib guided by NGS, serves as a prime example of this sequential approach. This outcome not only validates the strategy’s feasibility but also highlights the critical role of re-biopsy and molecular profiling at the time of progression. Treatment selection for patients with EGFR/ALK co-alterations is complex. There are no consensus guidelines regarding whether to prioritize EGFR-TKI, ALK-TKI, or combination strategies in the first-line setting. The decision is further complicated when PD-L1 expression is considered, as it strongly influences immunotherapy use. Nevertheless, in the presence of classical driver mutations, ICIs generally provide limited benefit, even with high PD-L1 expression. Unlike many reported cases of EGFR/ALK co-mutation, where EGFR-TKI monotherapy often fails to provide long-term control, this case illustrates that sequential therapy—starting with osimertinib followed by alectinib—can lead to a dramatic clinical response. The patient’s progression on osimertinib was accompanied by the persistence of the ALK fusion, which was identified as the main resistance driver. This emphasizes the importance of sequential therapy guided by molecular profiling.

This case demonstrates that the combination of EGFR exon 19 deletion, ALK V3a/b fusion, high PD-L1 expression, and the development of ERBB2 amplification poses a unique clinical challenge. While EGFR-TKI therapy was initially effective, the subsequent progression due to ERBB2 amplification highlights the importance of adapting the treatment approach based on evolving molecular profiles. This patient presented with high PD-L1 expression (TPS = 90%), a strong indication for immune checkpoint inhibitors (ICIs). However, it is well-established that patients with oncogene-driven tumors, such as those with EGFR mutations or ALK fusions, derive limited benefit from ICIs, even with high PD-L1 levels. Targeted therapy is the undisputed first-line choice in this setting due to significantly higher response rates. Although the patient exhibited high PD-L1 expression (TPS = 90%), immune checkpoint inhibitors (ICIs) were not selected as the treatment option due to the tumor’s oncogene-addicted nature. Tumors harboring classic driver mutations, such as EGFR mutations and ALK rearrangements, are primarily driven by persistent genetic alterations, which promote tumor growth. In these cases, ICIs generally offer limited clinical benefit, as the immune system is often unable to effectively recognize and attack the tumor cells. Moreover, studies have demonstrated that targeted therapies, such as EGFR-TKIs and ALK inhibitors, yield significantly higher response rates in these oncogene-driven tumors. While high PD-L1 expression is a strong indication for immunotherapy, the preferential choice in this case was targeted therapy due to its proven superior efficacy in tumors with defined oncogenic drivers like EGFR and ALK. Thus, although immunotherapy was considered, it was not prioritized in this case due to the clear advantage of targeted therapy in achieving better clinical outcomes. A real-world study (19) by Negrao et al. confirmed poor outcomes with ICIs across cohorts with various driver mutations (EGFR, ALK, HER2, etc.), irrespective of PD-L1 status. Intriguingly, some data suggest high PD-L1 expression may even predict primary resistance to EGFR-TKIs by inducing autophagy through the MAPK pathway, leading to a shorter PFS (20, 21). Conversely, analysis of the FLAURA trial demonstrated (22) that the clinical benefit of osimertinib was independent of PD-L1 status. Given these conflicting data, the role and timing of immunotherapy in this specific population are highly controversial. The therapeutic strategy for this patient adhered to the “target-first” principle, prioritizing targeted therapy plus chemotherapy over immunotherapy. The resulting 17-month period of disease control validates this approach. Further research is needed to clarify whether high PD-L1 expression functions as an independent prognostic marker or a mechanism of targeted therapy resistance in this context.

NSCLC is typically classified according to key driver genetic alterations such as EGFR mutations and ALK rearrangements. Recent studies have reported a growing recognition of NSCLC cases harboring both EGFR mutations and ALK rearrangements, although such co-occurrence remains relatively rare (23). To better contextualize the complexity of “triple-positive” NSCLC—defined by the coexistence of an EGFR mutation, an ALK rearrangement, and high PD-L1 expression—we have summarized key published cases in **Table 2**. These reports highlight the considerable heterogeneity in clinical presentation, therapeutic strategies, and clinical outcomes among patients with this molecular profile. The documented treatment regimens and corresponding clinical responses offer valuable insights for managing this rare and challenging NSCLC subset.

**Table 2:** Summary of clinical characteristics and treatment outcomes in NSCLC patients with co-occurring EGFR/ALK alterations and high PD-L1 expression.

Reference	Number of cases	EGFR mutation type	ALK rearrangement type	PD-L1 expression (TPS)	Treatment process
Hu et al. (2023) (17)	1 case	EGFR Exon 19 Deletion	EML4-ALK (V3)	High (TPS = 90%)	First-line: EGFR-TKI (Osimertinib) with chemotherapy. Disease progression observed after 17 months. Switched to ALK-TKI (Alectinib), leading to partial response.
Santelmo C, et al. (2013) (24)	Multiple cases	EGFR Exon 19 Deletion, L858R	ALK Rearrangement (EML4-ALK)	Not specified	Initial treatment with EGFR-TKI (Gefitinib or Erlotinib), disease progression, then switched to ALK-TKI (Crizotinib). Partial response observed.
Gaior JF, et al. (2013) (3)	58 cases (cohort)	EGFR L858R, Exon 19 Deletion	ALK Rearrangements (various)	Not specified	Retrospective analysis evaluating the impact of PD-1 inhibitors in patients with EGFR mutations and ALK rearrangements, showing low response rates.
Zhao et al. (2020) (18)	30 cases (retrospective)	Various EGFR Mutations	Various ALK Rearrangements	High (TPS = 80%-90%)	Multiple treatments: EGFR-TKI, ALK-TKI combination therapies. Sequential treatment with EGFR TKIs and crizotinib should be considered as a management option.
Zhang et al. (2020) (5)	1 case (retrospective)	EGFR (Exon 19 Deletion)	EML4‑ALK	Not specified	Erlotinib (EGFR‑TKI) resistance → progression → ALK‑TKI therapy (Crizotinib) started, partial response observed.

To place our findings within a broader evidence base, also synthesizes prominent published cases of triple-positive NSCLC. These examples illustrate the diversity in clinical characteristics and treatment outcomes, underscoring that—despite high PD-L1 expression—clinical decision-making often prioritizes targeted therapies such as EGFR tyrosine kinase inhibitors or ALK inhibitors. This reflects the nuanced therapeutic landscape for such patients. Further details of the summarized cases are provided in **Table 2**, titled “Summary of Clinical Features and Treatment Responses in NSCLC Patients with EGFR Mutations, ALK Rearrangements, and High PD-L1 Expression.

Acquired resistance to osimertinib was accompanied by the emergence of ERBB2 amplification. As a member of the ErbB receptor family, ERBB2 shares downstream pathways with EGFR, including PI3K/AKT/mTOR and RAS/MAPK (25). ERBB2 amplification leads to protein overexpression and the formation of dimers that drive constitutive downstream signaling, creating a bypass track that renders EGFR inhibition ineffective (26). This mechanism is well-documented, occurring in 5-10% of patients with osimertinib resistance (23, 27, 28). While potent anti-HER2 antibody-drug conjugates (ADCs) like trastuzumab-deruxtecan (T-DXd) are available, their strongest supporting data are in HER2-mutant NSCLC, with more limited evidence for tumors with ERBB2 amplification (29). Consequently, despite the detection of ERBB2 amplification, the clinical decision was not to immediately pivot to an anti-HER2 agent. Instead, guided by NGS data revealing the persistence of the ALK fusion as the primary druggable target, the strategy was to switch to alectinib. The profound response to alectinib validated this decision, demonstrating that targeting the dominant driver pathway can be highly effective even in the presence of multiple resistance mechanisms. While the ERBB2 amplification was not directly addressed, potent inhibition of the ALK pathway was sufficient to regain tumor control (30). While the patient initially responded to osimertinib in combination with chemotherapy, disease progression occurred after 17 months, revealing persistent ALK fusion and the emergence of ERBB2 amplification. The ALK V3a/b variant, which was present in this patient, is known to be associated with more aggressive disease and may contribute to resistance to targeted therapies, including EGFR-TKIs and ALK inhibitors. Specifically, V3a/b variants can alter the binding affinity of ALK inhibitors and impact downstream signaling pathways, potentially reducing the efficacy of treatments like osimertinib, which primarily targets EGFR mutations. Given that the ALK V3a/b variant is often linked with a more aggressive disease course, it may have contributed to the limited response to osimertinib and the rapid progression of disease. This variant could also have influenced the subsequent positive response to alectinib, an ALK inhibitor, as it may be more potent against certain ALK fusion variants. The superior efficacy of alectinib in this case may be related to its higher specificity and effectiveness against ALK fusion-driven tumors, particularly those with resistance mechanisms emerging from variants like V3a/b. Therefore, the dynamic nature of ALK fusion and its variants, including V3a/b, underscores the importance of molecular profiling at the time of disease progression to tailor subsequent therapies more effectively.

In conclusion, this case report details the successful management of a rare advanced NSCLC patient with concurrent EGFR 19-Del and EML4-ALK mutations. The initial treatment with osimertinib and chemotherapy, aligned with current standards, provided 76 weeks of disease control. Upon progression, NGS identified a persistent ALK fusion as the primary resistance driver, alongside acquired ERBB2 amplification. A strategic switch to second-line alectinib led to a rapid partial response, and the patient remains on treatment. This case highlights the critical importance of repeat molecular profiling upon disease progression to guide sequential targeted therapies and confirms that even in the presence of complex resistance mechanisms, identifying and inhibiting the dominant oncogenic driver can lead to excellent clinical outcomes (31).

## 4 Conclusion

This report presents an extremely rare case of advanced lung adenocarcinoma characterized by a triple-positive status: a canonical EGFR exon 19 deletion, a rare ALK (EML4-KIF5B V3a/b) fusion, and high PD-L1 expression (TPS = 90%). The key clinical takeaways from this case are as follows:

• Initial Management Strategy: For advanced NSCLC with co-existing EGFR and ALK driver mutations, for which no standard of care exists, an initial regimen of osimertinib combined with platinum-based chemotherapy achieved durable disease control for over 17 months. This demonstrates that a strategy targeting the presumed dominant driver gene (in this instance, EGFR) combined with chemotherapy is a feasible and effective initial therapeutic approach.
• Critical Role of Re-biopsy and NGS upon Progression: Following nearly 18 months of treatment, the patient experienced disease progression. A subsequent re-biopsy with NGS successfully elucidated the resistance mechanisms: the persistence of the original ALK fusion was identified as the primary driver of osimertinib resistance, likely in concert with a newly acquired ERBB2 amplification. This underscores the decisive role of repeat molecular profiling in guiding subsequent precision therapies after targeted treatment failure.
• Validation of Sequential TKI Therapy: Guided by the identified resistance mechanism, a timely switch to the potent ALK inhibitor alectinib resulted in a rapid and significant PR. This strongly validates the efficacy of a sequential TKI strategy (i.e., EGFR-TKI followed by ALK-TKI) as a highly effective salvage therapy for patients with dual driver mutations after resistance to one inhibitor develops.
• Prioritization of Targeted Therapy Over Immunotherapy: Despite exceptionally high PD-L1 expression, the first-line treatment prioritized a targeted therapy-based strategy over immunotherapy, which yielded an excellent clinical outcome. This case supports the current consensus that for NSCLC patients with well-defined classical driver mutations (e.g., EGFR, ALK), targeted therapy should remain the preferred first-line choice, even in the presence of high PD-L1 expression. The predictive value of PD-L1 and its relationship with targeted therapy resistance in this unique patient population warrant further investigation.

## Data Availability

The original contributions presented in the study are included in the article. Further inquiries can be directed to the corresponding author.
